# Redefining Prostate Cancer Precision: Radiogenomics, Theragnostics, and AI-Driven Biomarkers

**DOI:** 10.3390/cancers17233747

**Published:** 2025-11-24

**Authors:** Cristina Quicios Dorado, Ana Sánchez Ramírez, Marta Pérez Pérez, Manuel Saavedra Centeno, Lira Pelari Mici, Carlos Márquez Güemez, Eduardo Albers Acosta, Guillermo Celada Luis, Martin Costal, Patricia Toquero Diez, Nuria Romero Laorden, Raquel Jover Díaz, Clara Velasco Balanza, Luis San José Manso

**Affiliations:** 1 Department of Urology, Hospital Universitario de La Princesa, IIS Princesa, Universidad Autónoma de Madrid, 28006 Madrid, Spainclara.velasco@salud.madrid.org (C.V.B.); lsanjose.hcsc@salud.madrid.org (L.S.J.M.); 2 Department of Urology, Hospital Universitario del Henares, 28822 Madrid, Spain; asramirez@salud.madrid.org; 3 Department of Medical Oncology, Hospital Universitario de La Princesa, IIS Princesa, Universidad Autónoma de Madrid, 28006 Madrid, Spain; 4 Department of Nuclear Medicine, Hospital Universitario de La Princesa, IIS Princesa, Universidad Autónoma de Madrid, 28006 Madrid, Spain; raquel.jover@salud.madrid.org

**Keywords:** prostate cancer, PSMA, PET/CT, PET/MRI, theragnostics, radioligand therapy, radiogenomics, radiomics, liquid biopsy, ctDNA, circulating tumor cells, artificial intelligence, integrative models, personalized medicine

## Abstract

**Background/Objectives**: Prostate cancer is the most prevalent malignancy in men and remains a leading cause of cancer-related mortality worldwide. Conventional imaging modalities exhibit limited sensitivity, particularly in the context of disease recurrence and advanced disease. **Methods**: A narrative review was conducted of studies published between 2015 and 2025, identified through PubMed, Embase, and Cochrane. Eligible publications addressed advanced imaging techniques, PSMA-targeted diagnostics and therapies, radiogenomics, liquid biopsy approaches, and artificial intelligence applications and personalized medicine. Preclinical studies, single case reports, and conference abstracts without full text were excluded. **Results**: PSMA PET/CT outperforms conventional imaging for detection, and restaging, influencing clinical management across disease stages. Lutetium-177–PSMA-617 has become the standard radioligand therapy for metastatic castration-resistant prostate cancer, whereas alpha-emitting agents remain under clinical investigation. Radiogenomics and liquid biopsy assays (ctDNA, CTCs, AR-V7) provide complementary molecular insights. Artificial intelligence enhances imaging interpretations, standardization, and reproducibility, while multimodal data integration supports individualized risk stratification. Integrative models combining imaging, genomic, and liquid biopsy data pave the way toward precision oncology and personalized therapeutic decision-making. **Conclusions**: Advances in imaging and theragnostics are reshaping prostate cancer management, bridging the gap between molecular biology and clinical practice to enable precision oncology.

## Simple Summary

Prostate cancer is one of the most common cancers in men and a leading cause of cancer-related death. Traditional imaging methods such as CT, MRI, and bone scans often miss early recurrences and small metastases. Novel technologies are transforming this field. Molecular imaging with PSMA PET/CT provides superior accuracy for staging and restaging, while radioligand therapies enable simultaneous diagnosis and treatment (theragnostics). Liquid biopsy and artificial intelligence offer complementary tools for monitoring tumor biology and enhancing imaging interpretation. Together, these advances are driving prostate cancer management toward precision oncology, where decisions are tailored to each patient’s disease biology and clinical needs.

## 1. Introduction

According to the Global Cancer Observatory [1], prostate cancer (PCa) is the second most frequently diagnosed malignancy among men worldwide, with approximately 1.5 million new cases annually. PCa remains the most prevalent male cancer and a major cause of cancer-related mortality.

Conventional imaging modalities—computed tomography (CT), magnetic resonance imaging (MRI), and bone scintigraphy—have long constituted the diagnostic and staging cornerstone but exhibit limited sensitivity and specificity, particularly in early metastatic or recurrent disease [2].

Recent years have marked a paradigm shift driven by advances in molecular imaging, theragnostics, and computational technologies. Positron emission tomography (PET) using prostate-specific membrane antigen (PSMA)-targeted tracers has revolutionized disease detection and treatment guidance across all disease stages. Beyond imaging, PSMA-targeted radioligand therapies, such as lutetium-177–PSMA-617 (^177^Lu-PSMA-617), and actinium-225–PSMA (^225^Ac-PSMA), have introduced a novel theragnostic model linking molecular diagnosis and therapy [3,4,5].

Multiparametric MRI (mpMRI), hybrid PET/MRI, and artificial intelligence (AI)-assisted analysis are further refining local staging and risk stratification. The integration of radiomics, radiogenomics, and liquid biopsy provides complementary molecular insights, enabling more accurate characterization of tumor biology and therapeutic resistance.

This review summarizes current evidence on advanced imaging and theragnostic strategies in prostate cancer, highlighting the role of molecular radiotracers, PSMA-targeted therapies, integrative imaging–biomarker approaches, and AI-driven tools in advancing precision oncology, supported by emerging integrative models that enable personalized risk assessment and therapeutic decision-making.

## 2. Materials and Methods

This work was designed as a narrative review focused on advances in imaging and theragnostic strategies for prostate cancer. We performed a comprehensive literature search in PubMed/MEDLINE, Embase, and the Cochrane Library, covering the period from January 2015 to September 2025. The search strategy combined terms related to prostate cancer with keywords including “PSMA”, “PET/CT”, “PET/MRI”, “radioligand therapy”, “theragnostics”, “radiomics”, “radiogenomics”, “liquid biopsy”, “ctDNA”, “circulating tumor cells”, “biomarkers”, “artificial intelligence”, “integrative models” and “personalized medicine”.

Eligible studies included original clinical research, systematic reviews, meta-analyses, and pilot feasibility studies assessing diagnostic, prognostic, or therapeutic applications of advanced imaging modalities and theragnostic approaches in prostate cancer. Preclinical studies, case reports, editorials, commentaries, and conference abstracts without full text were excluded.

Two independent reviewers screened titles and abstracts, followed by full-text evaluation of potentially relevant publications. Discrepancies were resolved by consensus. Extracted data included study design, patient population, imaging modality or biomarker assessed, therapeutic intervention (if applicable), and main clinical outcomes such as sensitivity, specificity, detection rate, staging accuracy, management changes, treatment response, and survival endpoints.

Given the heterogeneity of study designs and outcome measures, results were synthesized narratively. Particular attention was given to the integration of PSMA PET with conventional imaging, the role of radioligand therapies, the contribution of radiogenomics and liquid biopsy, and the emerging utility of artificial intelligence in imaging interpretation and decision support.

To ensure methodological transparency, the review was structured around clearly defined clinical questions. These were focused on the role of advanced imaging, theragnostic approaches, and biomarker integration in prostate cancer management. Table 1 summarizes the scope of the review, including population, interventions, comparisons, outcomes, study types, and search strategy.

## 3. Results

### 3.1. Molecular Imaging

Molecular imaging has revolutionized the diagnostic and therapeutic landscape of prostate cancer by providing superior sensitivity, specificity, and biological characterization compared with conventional imaging modalities [2]. Traditional anatomical techniques such as CT, MRI, and bone scintigraphy offer mainly morphological information, whereas radionuclide-based approaches—namely PET and single-photon emission computed tomography (SPECT)—enable disease visualization at the molecular level. These modalities can depict functional alterations such as changes in perfusion, metabolism, cellular proliferation, and, most importantly, expression of prostate-specific molecular targets such as PSMA [3,4]. Although PET and SPECT lack inherent anatomical detail, their integration with CT or MRI substantially enhances diagnostic accuracy. Among available molecular imaging agents, PSMA PET has shown clear superiority over conventional imaging for both primary staging and detection of biochemical recurrence. This has led to significant modifications in clinical decision-making and is now endorsed by contemporary international guidelines as the preferred imaging modality across multiple disease stages [5].

#### 3.1.1. Radiotracers

Radiotracers are central to prostate cancer imaging, enabling molecular characterization and disease localization beyond the limits of conventional modalities. Several PET agents are now established in clinical use, while others remain under investigation. The most clinically relevant are summarized in Table 2.

##### Prostate-Specific Membrane Antigen (PSMA) Ligands

PSMA is a type II transmembrane glycoprotein markedly overexpressed in prostate adenocarcinoma relative to benign tissue, with expression levels increasing in higher-grade, castration-resistant, and metastatic disease. Its extracellular domain provides an accessible target for radiolabeled small-molecule ligands used in PET imaging [6]. The main clinical tracers—^68^Ga-PSMA-11, ^18^F-DCFPyL, and ^18^F-PSMA-1007—exhibit high affinity for PSMA and have consistently demonstrated superior sensitivity and specificity over conventional imaging in both initial staging and biochemical recurrence [2].

^68^Ga-PSMA-11 and ^18^F-DCFPyL have similar lesion detection rates and biodistribution, both are primarily excreted via the urinary tract, which can obscure pelvic lesions due to bladder activity. ^18^F-PSMA-1007 is mainly excreted hepatobiliary, resulting in minimal urinary bladder activity and improved visualization of pelvic lesions, but it is associated with a higher rate of benign bone uptake, leading to more equivocal findings and potential false positives in bone. All three tracers are effective for prostate cancer imaging, but selection should consider excretion profile and interpretive pitfalls [7,8]. Beyond diagnosis, PSMA-based imaging underpins theragnostic approaches, guiding selection and response assessment for ^177^Lu-PSMA-617 and ^225^Ac-PSMA-617 radioligand therapies in metastatic castration-resistant prostate cancer (mCRPC) [4,9].

##### Amino Acid Analogues

^18^F-fluciclovine (anti-^18^F-FACBC) targets upregulated amino acid transport in malignant cells. FDA-approved for detecting recurrent prostate cancer, it demonstrates superior performance to choline tracers, particularly at intermediate PSA levels [3,9]. However, sensitivity remains lower than that of PSMA-based PET, particularly when PSA < 0.5 ng/mL [10].

##### Choline-Based Tracers

^11^C-choline and ^18^F-choline were the first PET tracers widely adopted for prostate cancer, exploiting increased phospholipid synthesis in proliferating cells. Although still used in Europe and Japan, especially where PSMA tracers are unavailable, their diagnostic yield is inferior [3].

The diagnostic sensitivity of choline-based PET tracers decreases notably at PSA levels below 2 ng/mL and becomes particularly limited when PSA falls below 1 ng/mL, reducing their effectiveness for detecting recurrent or metastatic disease in early biochemical relapse [11].

##### Bone-Specific Tracers

^18^F-sodium fluoride (NaF) provides high-resolution skeletal imaging by incorporating into hydroxyapatite at sites of bone turnover. It shows excellent sensitivity and specificity for detecting osseous metastases but is limited to bone assessment and has been largely replaced by PSMA PET/CT, which simultaneously evaluates bone, soft-tissue, and nodal disease. However, its specificity is limited, as it cannot differentiate metastatic lesions from benign conditions like bone degeneration or inflammation, potentially resulting in false positives [3].

##### ^18^F-Fluorodeoxyglucose

^18^F-fluorodeoxyglucose (FDG) has limited value in typical prostate adenocarcinoma due to low glycolytic activity but remains useful in aggressive, poorly differentiated, or neuroendocrine variants. FDG PET may complement PSMA PET in advanced or dedifferentiated disease phenotypes [4,9].

#### 3.1.2. PSMA Pitfalls

Although PSMA PET/CT provides good sensitivity for detecting prostate cancer, it is also susceptible to several interpretive pitfalls. Normal physiological uptake of PSMA-targeted tracers occurs in organs such as the salivary and lacrimal glands, liver, spleen, kidneys, and small intestine, as well as in parasympathetic ganglia like the celiac or stellate ganglia. These findings can resemble metastatic deposits if their characteristic low-grade, bilateral, or linear distribution is not recognized. In addition, the strong urinary excretion of the tracer may produce artifacts or obscure pelvic lesions near the bladder or ureters. Uptake can also appear in benign conditions—such as healing fractures, degenerative bone disease, Paget disease, fibrous dysplasia, or inflammatory processes—leading to potential false-positive interpretations [6,12]. PSMA expression in the neovasculature of non-prostatic tumors, such as renal cell carcinoma, hepatocellular carcinoma, or glioblastoma, can produce misleading uptake unrelated to prostate cancer [12]. For these reasons, careful correlation with anatomic imaging and clinical context is essential to accurately distinguish physiological or benign uptake from true metastatic disease.

False-negative results can also occur, particularly in prostate cancer subtypes with little or no PSMA expression, including poorly differentiated or neuroendocrine tumors, or in lesions that have lost expression after extensive treatment. Furthermore, PSMA PET/CT may also miss nodal or visceral metastases smaller than 5 mm [6,12].

#### 3.1.3. PSMA PET in Prostate Cancer Staging

PSMA PET/CT is significantly more accurate than conventional imaging (CT and bone scintigraphy) for detecting pelvic nodal and distant metastatic disease in men with high-risk prostate cancer. The proPSMA trial [2] is a phase 3, multicenter, randomized clinical study comparing conventional imaging techniques with ^68^Ga-PSMA PET/CT in patients with localized high-risk prostate cancer who were candidates for curative-intent treatment. PSMA PET/CT achieved an accuracy of 92% compared to 65% for conventional imaging, with higher sensitivity (85% vs. 38%) and specificity (98% vs. 91%) for both nodal and distant metastases. PSMA PET/CT also resulted in fewer equivocal findings (7% vs. 23%) and led to more frequent changes in clinical management (28% vs. 15%) than conventional imaging. Additionally, PSMA PET/CT was associated with lower radiation exposure.

Several meta-analyses and systematic reviews corroborate the findings of the proPSMA trial. Wu et al. [13] analyzed 13 studies including 1597 patients with intermediate- or high-risk prostate cancer scheduled for radical prostatectomy. ^68^Ga-PSMA PET/CT demonstrated higher sensitivity (0.65 vs. 0.41) and comparable specificity (0.94 vs. 0.92) for detecting lymph node metastases compared with MRI. Another meta-analysis [14] including 2.431 patients with intermediate- to high-risk prostate cancer, showed that ^68^Ga-PSMA PET/CT had greater sensitivity and specificity than mpMRI (73.7% vs. 38.9%; 97.5% vs. 82.6%) and CT (73.2% vs. 38.5%; 97.8% vs. 83.6%) for nodal staging. The low sensitivity of MRI in detecting lymph node metastases may be attributed to its reliance on morphological criteria and nodal size. MRI is typically capable of identifying lymph nodes measuring 8–10 mm, whereas approximately 80% of metastatic nodes in prostate cancer fall below this threshold. Regarding the type of ligand, ^68^Ga-PSMA has shown higher sensitivity than ^18^F-PSMA for nodal staging. One of the main limitations of PSMA PET imaging is its inability to reliably detect micrometastatic deposits smaller than 3 mm, which often fall below the spatial resolution of the modality [5]. Furthermore, PSMA PET/CT has demonstrated higher accuracy than conventional imaging for the detection of bone metastases, with superior sensitivity and specificity (98% vs. 73% and 96.2% vs. 79.1%, respectively). In addition, PSMA PET overcomes the limitations of bone scintigraphy in identifying lytic lesions and reduces the risk of false-positive findings, thereby minimizing overtreatment of non-existent metastatic disease [14].

^68^Ga PSMA PET/CT shows lower sensitivity and specificity than both PSMA PET/MRI and multiparametric MRI in detecting extraprostatic extension and seminal vesicle invasion. MRI remains the most specific technique for local staging. The relative inferiority of PSMA PET/CT may be related to lower tracer uptake within the primary tumor and to variations in bladder filling, which can obscure or reduce accuracy in the evaluation of seminal vesicle involvement. These findings indicate that, in local tumor assessment—where precise delineation of primary tumor extension critically depends on high-resolution anatomical detail—the spatial resolution of multiparametric MRI cannot be replaced, although it can be complemented by the molecular sensitivity of PSMA PET for detecting small or subtle lesions [14].

^18^F-PSMA-1007 PET/CT has also demonstrated superior accuracy compared with multiparametric MRI for locoregional staging of prostate cancer. In men with intermediate- and high-risk disease undergoing radical prostatectomy, ^18^F-PSMA-1007 PET/CT more accurately identified the final pathological tumor stage (45% vs. 28%), dominant nodule localization (94% vs. 83%), laterality (64% vs. 44%), and extracapsular extension (75% vs. 63%), supporting its integration into preoperative assessment of intermediate- and high-risk prostate cancer [15].

According to some studies [16,17], the intensity of intraprostatic PSMA uptake (SUVmax) reflects underlying tumor aggressiveness, showing strong correlations with PI-RADS score and histologic grade at diagnosis, and acting as an independent predictor of biochemical recurrence after radical prostatectomy, even when adjusted for PSA level, Gleason score, and pathological stage. Furthermore, higher SUVmax values exhibit excellent specificity for clinically significant prostate cancer, supporting the use of PSMA uptake as a noninvasive imaging biomarker of tumor biology that complements multiparametric MRI in preoperative risk assessment and treatment planning, with a median PSMA SUVmax of 4 (IQR: 3.4–5.1) when no cancer was found on biopsy, versus 12.3 (IQR: 6.3–15.6) for ISUP grade group 5 malignancy [16].

Accurate nodal staging remains a central but controversial aspect of the surgical management of high-risk prostate cancer. While pelvic lymph node dissection (PLND) provides histopathologic confirmation, it carries procedural morbidity and uncertain oncologic benefit. Recent multicenter trials have evaluated PSMA PET/CT as a noninvasive alternative. In both the ^68^Ga-PSMA-11 and ^18^F-DCFPyL prospective phase 3 studies, sensitivity for nodal metastasis detection compared with extended PLND ranged between 30–40%, whereas specificity consistently exceeded 95%, indicating that a positive PSMA PET finding is highly predictive of true nodal disease, but a negative result does not exclude microscopic metastases [18,19,20]. A meta-analysis [21] including over 2800 patients reported pooled sensitivity and specificity of 58% and 95%, respectively, with a negative predictive value of 87% on a per-patient basis and 97% per node. These data suggest that although PSMA PET/CT provides excellent specificity and can guide surgical extent, its limited sensitivity in detecting subcentimeter metastases precludes its use as a definitive substitute for PLND in high-risk patients.

#### 3.1.4. PSMA PET in Biochemical Recurrence

PSMA PET has emerged as the preferred imaging modality for evaluating biochemical recurrence (BCR) of prostate cancer owing to its superior sensitivity and specificity compared with conventional imaging techniques such as CT, MRI, and bone scintigraphy—particularly at low PSA levels.

In the updated systematic review and meta-analysis by Perera et al. [22], which included 4790 patients across 37 studies, the detection rate of ^68^Ga-PSMA PET/CT increased proportionally with serum PSA. The proportion of positive scans was 33% for PSA < 0.2 ng/mL, 45% for 0.2–0.49 ng/mL, 59% for 0.5–0.99 ng/mL, 75% for 1–1.99 ng/mL, and 95% for ≥2 ng/mL. On pooled analysis, PSMA PET demonstrated high diagnostic accuracy, with per-patient sensitivity of 77% and specificity of 97%, confirming its superior performance over standard imaging and choline-based PET tracers.

In a more recent and comprehensive systematic review and meta-analysis [23], including 8119 patients with BCR after radical prostatectomy or radiotherapy, PSMA PET/CT demonstrated high detection rates for all sites of recurrence—local, nodal, and distant—even at low PSA levels. The pooled positivity rate was 48% for PSA 0.2–0.49 ng/mL, increasing to 66% for PSA 0.5–0.99 ng/mL and exceeding 90% for PSA > 2 ng/mL. Detection rates were consistently higher after radiotherapy than after prostatectomy (92% vs. 60%), largely reflecting differences in PSA thresholds at imaging (median PSA 5.8 ng/mL vs. 0.45 ng/mL). Anatomically, positivity rates were 23% for local recurrence, 32% for pelvic nodes, 16% for bone metastases, and 1% for visceral disease.

The clinical impact of PSMA PET In BCR is well established, with multiple prospective studies demonstrating a significant influence on therapeutic decision-making. Across trials, management modification occurs in 30–60% of patients, typically involving treatment escalation, refined radiotherapy targeting, or avoidance of unnecessary systemic therapy [23,24]. In large prospective cohorts such as the Ontario PREP registry and the IAEA-PSMA study, PSMA PET identified recurrence in approximately two-thirds of patients and altered management in more than half [24,25]. PSMA PET is also valuable for identifying oligometastatic disease, guiding metastasis-directed therapy, and individualizing salvage treatment strategies [26,27].

Randomized data further support its integration into salvage therapy. In the EMPIRE-1 trial [28] incorporation of ^18^F-fluciclovine PET/CT into post-prostatectomy salvage radiotherapy (SRT) planning improved 3-year event-free survival from 63% to 75.5% (*p* = 0.0028), demonstrating that molecular imaging–guided planning could translate into superior oncologic control. However, although molecular imaging can refine treatment selection and planning, the oncologic benefit of PSMA PET–guided management remains unproven, and further randomized outcome studies are required to confirm its therapeutic value.

#### 3.1.5. Radiopharmaceuticals—Theragnostics

Radioligand therapies (RLT) (such as radium-223 dichloride and Lutetium-177 (^177^Lu)-PSMA 617) can target prostate cancer cells while sparing most normal tissues in patients who have been selected with the use of imaging to confirm radionuclide binding. Castration-resistant prostate cancer is the disease stage in which this therapeutic approach has been most extensively developed.

Alpha-emitting radiopharmaceuticals, most notably radium-223 dichloride, represent an established therapeutic option in mCRPC with symptomatic bone metastases and no visceral disease. Radium-223 is a bone-targeted alpha emitter that acts as a calcium mimetic, selectively accumulating in regions of increased osteoblastic activity where it emits high-linear-energy-transfer (LET) alpha particles with a path length below 100 µm, inducing irreparable double-strand DNA breaks in adjacent tumour cells while minimizing marrow and soft-tissue toxicity [29].

In the pivotal ALSYMPCA trial [30], radium-223 demonstrated clear oncologic benefit in patients with castration-resistant prostate cancer and symptomatic bone metastases, who failed or were unfit for docetaxel. Ra-223 improved overall survival, delaying the onset of symptomatic skeletal events, and enhancing quality of life, with a favourable safety profile and minimal myelosuppression. Subsequent studies have explored combination strategies: the ERA-223 trial [31] combining radium-223 with abiraterone acetate and prednisone did not improve efficacy and was associated with an increased risk of fractures in patients not receiving bone-protective agents, while early-phase investigations combining radium-223 with the immune checkpoint inhibitor pembrolizumab [32] suggest potential synergistic immunomodulatory activity, though clinical benefit remains unproven.

^177^Lu-PSMA-617 is a small-molecule PSMA ligand chelated to lutetium-177 that internalizes upon binding to cell-surface PSMA and delivers beta-particle radiation (medium LET, millimeter-range path length) selectively to PSMA-positive cells and surrounding microenvironment, enabling cross-fire to heterogeneous foci while limiting exposure to normal tissues [33]. Patient selection typically requires ^68^Gallium-labelled PSMA-positive PET (and exclusion of PSMA-negative/FDG-discordant disease in some trials).

In the phase 3 VISION trial [34,35], conducted in patients with mCRPC previously treated with an androgen receptor pathway inhibitor (ARPI) and one or two taxane regimens, the addition of ^177^Lu-PSMA-617 to standard of care significantly improved both overall survival and radiographic progression-free survival compared with standard therapy alone, with a manageable safety profile characterized mainly by fatigue, anemia, xerostomia, and mild cytopenias [35,36,37,38]. Similarly, in the TheraP [39] randomized phase 2 trial, ^177^Lu-PSMA-617 demonstrated a higher PSA response rate and longer progression-free survival compared with cabazitaxel in post-docetaxel and androgen-receptor pathway inhibitor mCRPC, with no differences in overall survival between both groups.

Combination therapies are currently under investigation. Specifically, the EnzaP trial, a randomized phase II study evaluating enzalutamide in combination with ^177^Lu-PSMA-617 in mCRPC patients, has demonstrated an improvement in median PSA progression-free survival compared with enzalutamide alone [40].

PSMA expression increases upon prostate tumorigenesis, and a further increase was noted with a higher Gleason score and along cancer progression. Moreover, high PSMA expression has been associated with poorer clinical outcomes. But significant interpatient, intertumoral and intratumoral heterogeneity of PSMA expression has been observed in prostate cancer. The therapeutic efficacy of PSMA-targeted radioligand therapy depends on the level and uniformity of PSMA expression across tumor sites [41]. Expression of the FOLH1 gene, which encodes PSMA, is dynamically regulated by androgen receptor (AR) signaling—it is upregulated by androgen deprivation but downregulated after prolonged AR pathway inhibition or in AR-independent tumor evolution. Tumors that undergo lineage plasticity toward a neuroendocrine phenotype, often associated with loss of RB1 and TP53 and activation of MYCN and AURKA, typically show reduced or absent PSMA expression, leading to limited ligand uptake and therapeutic resistance. From a clinical perspective, these biological mechanisms translate into variable treatment responses. Intrapatient heterogeneity, reflected by PSMA-negative or FDG-positive lesions, identifies resistant subclones unlikely to benefit from PSMA-RLT. Conversely, transcriptional regulators such as HOXB13 may enhance FOLH1 transcription, suggesting potential strategies to modulate PSMA expression and optimize patient selection.

Alpha-emitting PSMA radioligands, particularly ^225^Ac-PSMA-617, represent the next generation of targeted radionuclide therapy for mCRPC. Actinium-225 emits high LET α-particles with an extremely short path length (<100 µm), producing dense, clustered double-strand DNA breaks and potent cytotoxicity within individual tumor cells while sparing surrounding tissue [41,42]. Compared with β-emitting ^177^Lu-PSMA-617, ^225^Ac-PSMA delivers far higher energy deposition per track, enabling activity in low-volume or heterogeneous PSMA-expressing disease, where cross-fire from β-emitters may be insufficient. However, treatment is limited by salivary-gland and lacrimal-gland toxicity (xerostomia, keratoconjunctivitis), marrow suppression at cumulative doses, and challenges in dose standardization and isotope supply.

Early-phase and compassionate-use series in heavily pretreated mCRPC have shown high biochemical response rates (PSA declines in 60–80%) and encouraging radiographic disease control, including in patients previously refractory to ^177^Lu-PSMA. Current clinical trials are evaluating ^225^Ac-PSMA-617 both as salvage monotherapy after β-therapy and in earlier treatment lines, including combinations with androgen-receptor inhibitors or DNA-damage-response modulators [33,36].

RLT is the most established in mCRPC, but there are two additional PSMA-targeted therapeutic strategies in development [41]:

- Antibody–drug conjugates (ADCs):
  These consist of anti-PSMA monoclonal antibodies linked to cytotoxic agents. First-generation ADCs (e.g., MLN2704, PSMA-ADC, MEDI3726) achieved modest antitumor activity but were constrained by dose-limiting toxicities such as neuropathy and mucocutaneous effects. Current research focuses on small-molecule drug conjugates and optimized linkers to enhance selectivity and safety.

- PSMA-directed CAR-T cell therapy:
  PSMA-targeted CAR-T cells involve autologous T lymphocytes engineered to recognize PSMA on prostate cancer cells. Early trials in metastatic castration-resistant disease have shown PSA and radiologic responses in a subset of patients, confirming antitumor activity. However, efficacy is limited by cytokine-release syndrome, T-cell exhaustion, and antigen heterogeneity. Current efforts aim to improve persistence and safety through dual-target designs and checkpoint inhibitor combinations.

- PSMA-targeted bispecific T-cell engagers:
  Bispecific T-cell engagers (BiTEs) simultaneously bind PSMA on tumor cells and CD3 on T cells, triggering direct cytotoxicity. Agents such as AMG 212 and AMG 160 have demonstrated measurable PSA responses in advanced disease, but cytokine-release syndrome and dosing challenges remain major limitations. Ongoing trials seek to optimize pharmacokinetics and immune tolerability to enhance clinical applicability.

Regarding metastatic hormone-sensitive prostate cancer and in the bone-targeted setting, the RAVENS phase II trial [43] evaluated radium-223 in combination with stereotactic ablative radiotherapy (SABR) for oligometastatic castration-sensitive prostate cancer. The study showed that adding radium-223 did not improve progression-free survival over SABR alone, though it identified genomic and immunologic correlates of outcome, such as high-risk DNA repair gene alterations (ATM, BRCA1/2, RB1, TP53) and reduced T-cell receptor diversity as poor prognostic features. Similarly, the Rad-SABR trial [44] suggested that SABR remains the mainstay for consolidating limited metastatic lesions, while the benefit of adding alpha-emitters in this low-volume, hormone-sensitive context remains unproven.

Ongoing phase 3 trials are assessing ^177^Lu-PSMA-617 in earlier disease settings, including pre-taxane mCRPC and hormone-sensitive metastatic prostate cancer (mHSPC), to evaluate whether earlier use can improve survival and delay progression compared with standard therapy [33].

Early studies are assessing ^177^Lu-PSMA-617 in oligometastatic and hormone-sensitive prostate cancer, showing preliminary signals of delayed progression and deferred androgen deprivation with good tolerability [45].

Beyond radionuclide-based theragnostics, RNA-directed strategies are emerging as a complementary experimental approach in metastatic castration-resistant prostate cancer (mCRPC). MicroRNA replacement—particularly miR-34a, a tumour-suppressive regulator frequently downregulated in TP53-altered CPRC—has shown the capacity to inhibit oncogenic pathways, stemness programs, and treatment resistance. Recent preclinical work [46] demonstrates that targeted delivery systems can overcome the major barriers to miRNA therapy, including instability, limited tumor uptake, and endosomal sequestration. Conjugating miR-34a to PSMA-directed ligands, combined with endosomal escape enhancers and chemical stabilization, enabled selective intratumoral accumulation and significant growth inhibition in PSMA-positive mCRPC models, highlighting a biologically rational complement to PSMA-based RLT. In contrast, folate-conjugated miR-34a lacked efficacy in prostate cancer owing to absent FOLR1 expression, underscoring the necessity of tumor-specific targeting ligands for clinical translation [47].

### 3.2. Multiparametric Imaging and Artificial Intelligence (AI)

#### 3.2.1. Magnetic Resonance Imaging in Initial Staging

mpMRI plays a central role in the initial staging of prostate cancer by combining anatomical and functional imaging to evaluate tumor location, local extension, and risk stratification. mpMRI, interpreted using the Prostate Imaging Reporting and Data System (PI-RADS), is recommended for all men with suspected clinically significant prostate cancer (csPCa), including biopsy-naïve patients and those with prior negative biopsies [48]. mpMRI shows high sensitivity (~85%) for detecting csPCa and provides essential information for assessing extracapsular extension and seminal vesicle invasion, crucial for treatment planning [48,49,50]. MRI-targeted biopsy with mpMRI improves csPCa detection and reduces unnecessary procedures compared with systematic biopsy alone [48,51]. Biparametric MRI (bpMRI) is being investigated as a faster, lower-cost alternative, though mpMRI remains the standard for local staging [49].

MRI also plays a role in risk stratification and selection for active surveillance (AS), as lesion visibility and PI-RADS score correlate with tumor aggressiveness [51,52]. However, limitations include inter-reader variability and the inability to completely exclude csPCa in patients with negative or equivocal MRI findings, necessitating continued use of systematic biopsy in some cases [48,51].

In nodal staging, mpMRI shows lower sensitivity than PSMA PET for detecting pathological lymph nodes, while specificity varies between studies—being similar in some analyses [13] and slightly inferior in others [14]. These differences arise from MRI’s reliance on morphologic criteria and lesion size, whereas PSMA PET detects molecular tracer uptake, allowing identification of smaller metastatic deposits with greater accuracy.

#### 3.2.2. PET/MRI

In the initial staging setting, PET-MRI—particularly with PSMA-targeted tracers—demonstrates high sensitivity for primary tumor detection (pooled sensitivity ~95%) and is effective for local staging, including assessment of extraprostatic extension and seminal vesicle invasion, as well as nodal and bone metastases. The addition of PET to MRI improves detection rates for both local and distant disease, and the hybrid approach can reduce interreader variability and increase diagnostic confidence compared to MRI or PET alone [53]. In a meta-analysis comparing PSMA PET to conventional imaging modalities in initial staging of prostate cancer PSMA PET-MRI was significantly more sensitive than MRI alone for extraprostatic extension and seminal vesicle invasion, while PSMA PET/CT was less sensitive than MRI alone [14].

The PRIMARY study [16] found that the combination of PSMA and MRI had a higher negative predictive value compared to MRI alone (91% vs. 83%, *p* < 0.001), with higher sensitivity (97% vs. 83%, *p* < 0.001) but lower specificity (40% vs. 53, *p* = 0.011). Therefore, combined imaging could reduce the number of unnecessary biopsies in men with negative PET-MRI. The ongoing PRIMARY2 trial [54] aims to assess the utility of the addition of PSMA PET/CT to equivocal (PI-RADS 3) or negative (PI-RADS 2 with red flags) mpMRI, in order to avoid unnecessary prostate biopsies.

In the context of BCR, PET-MRI offers complementary strengths: the MRI component is particularly sensitive for detecting local recurrence in the prostate bed, especially at lower PSA levels, while the PET component is superior for identifying nodal and distant metastases [55,56]. Combined PET-MRI assessment improves overall tumor localization and staging accuracy, with a sensitivity for detecting prostate cancer in BCR varying between 68% and 80% [55,56].

Several studies show that the addition of PET to MRI or CT can change intended management in up to half of patients with BCR, although a survival benefit from this management change has yet to be proven in further studies [57,58]. Detection rates increase with higher PSA, but PET-MRI remains effective even at low PSA thresholds [58].

#### 3.2.3. Artificial Intelligence (AI)

AI is rapidly transforming prostate cancer detection and diagnosis, particularly through the application of deep learning and machine learning to mpMRI and advanced imaging modalities. AI models have demonstrated high accuracy for identifying csPCa, often matching or exceeding the performance of radiologists, and are increasingly validated in both retrospective and prospective multicenter studies [59,60,61,62,63,64].

AI-based models, such as XGBoost, random forest, and neural networks, use machine learning algorithms to integrate radiological features (e.g., PI-RADS scores) with clinical variables (age, PSA, prostate volume, PSA density, digital rectal examination (DRE) findings, family history, prior biopsy) to predict clinically significant prostate cancer (csPCa) and reduce unnecessary biopsies. XGBoost builds sequential decision trees that correct prior errors to improve accuracy; random forest aggregates multiple randomized trees for robust predictions; and neural networks model complex nonlinear relationships, excelling at pattern recognition in imaging data [59].

In a large cohort, the XGBoost machine learning model outperformed traditional logistic regression in predicting clinically significant prostate cancer (csPCa). At a 90% sensitivity, it achieved a specificity of 64%, indicating that it could avoid more than 40% of unnecessary biopsies while maintaining a 10% miss rate for csPCa detection [59]. Model interpretability using SHAP values highlight PI-RADS, DRE, and family history as key predictors [59].

Advanced imaging metrics, such as diffusion basis spectrum imaging (DBSI), further enhance AI model performance. A DBSI-based AI model independently predicted csPCa and, when combined with PI-RADS, achieved an area under the curve (AUC) of 0.894, enabling a reduction in unnecessary biopsies by 27% while missing only 2% of csPCa [60]. Deep learning algorithms trained on biparametric or multiparametric MRI have shown comparable sensitivity and specificity to expert radiologists, with some models improving positive predictive value and specificity, especially when used to adjudicate indeterminate (PI-RADS 3) lesions [61,62,63,64].

Explainable AI (XAI) models provide visual and textual justifications for their predictions, improving nonexpert confidence and reducing reading time, while maintaining high sensitivity for csPCa [61]. Human-in-the-loop AI systems, such as those based on PI-RADS, can match or outperform general radiologists and offer diagnostic benefits in settings lacking subspecialist expertise [65].

A large international diagnostic study (PI-CAI Consortium) [66] demonstrated that AI-assisted interpretation of prostate MRI significantly improves the detection of clinically significant prostate cancer compared to unassisted readings. Specifically, AI assistance increased the area under the receiver operating characteristic curve by 3.3%, with improvements in both sensitivity and specificity at the PI-RADS ≥ 3 threshold. Notably, the benefit was more pronounced among nonexpert readers, suggesting AI can help standardize performance across varying levels of radiologist experience and potentially improve access to high-quality prostate MRI interpretation globally [67].

AI-based radiomics and multi-omics models are being developed for risk stratification, tumor grading, and prediction of treatment response, leveraging quantitative imaging features and clinical data [68,69]. Bimodal models that integrate imaging and clinical data improve both diagnostic performance and prediction reliability, with uncertainty quantification enhancing clinical applicability [69]. AI also supports automated lesion segmentation and grading, with convolutional neural networks and cascaded deep learning algorithms achieving high detection rates for csPCa and reducing inter-reader variability [37,38].

### 3.3. Radiogenomics

Radiomics involves the extraction of quantitative features from radiologic images that are imperceptible to radiologists, using mathematical analysis or, more recently, AI. Its main goal in PCa is to differentiate clinically significant PCa from indolent PCa, aiming to improve the predictive performance of the PI-RADS in detecting csPCa and thereby avoid unnecessary biopsies [70].

On the other hand, genomics is the study of entire genomes, including genetic elements. Genomics combines recombinant DNA, DNA sequencing methods, and bioinformatics to sequence, assemble, and analyze the structure and function of genomes [70,71]

Radiogenomics is an interdisciplinary field that integrates radiologic data (radiomic analysis) with genomic information from tumor tissue and could serve as a biomarker of disease. Its main objective is to establish quantifiable correlations between visual phenotypes extracted from imaging—such as textural features from MRI or CT scans—and molecular alterations in the tumor. These include mutations, individual genes, relevant molecular pathways (such as PI3K, MYC, hypoxia), clinical transcriptomic signatures (e.g., Decipher, Oncotype Dx, Prolaris, PORTOS), and genomic events such as PTEN loss, ERG fusion, and copy number alterations (CNAs) [70,71,72].

This integration allows the development of predictive models that can reflect the biological behavior of cancer, support risk stratification, anticipate treatment response, and guide personalized clinical decision-making.

In recent years, multiple studies have explored the applicability of radiogenomics in PCa. Ogbonnaya CN et al. [72] developed a radiogenomic map using pre-biopsy biparametric MRI (T2WI and ADC) and genetic profiles obtained through DNA sequencing in patients with localized PCa. Their goal was to evaluate the correlation between molecular and imaging phenotypes in localized PCa, using histopathology from radical prostatectomy as the reference. In their analysis, radiomic features significantly correlated with apoptosis-related genotypes (TP53BP2, BAG3, TRAIL.R2), hypoxia (P4HA1, ANGPTL4, VEGFA), and androgen receptor expression (NKX3.1, KLK2, KLK3). This correlation varied by Gleason grade, and the radiogenomic model achieved an AUC of 0.95 for predicting csPCa, outperforming models based on imaging or genomics alone.

Radiogenomics has also been used to relate quantitative imaging features (mpMRI) and transcriptomic characteristics (RNA sequencing) of PCa to identify biomarkers of tumor aggressiveness [73]. Specifically, Dinis Fernandes et al. [73] used texture features from all mpMRI sequences (T2W, DWI, DCE), combined with pharmacokinetic maps derived from DCE images, obtained from magnetic resonance dispersion imaging (MRDI). For transcriptomic data, they used transcription factors (TFs; 17 genes, including STAT6, TFAP2A, ETS2), signaling pathways (10, including PI3K, mTOR, p53, hypoxia), and clinical transcriptomic signatures (Decipher, PORTOS, Oncotype DX). They developed machine learning models to classify tumors as clinically significant (ISUP ≥ 3) or not, validated in an independent cohort. Strong and significant correlations were observed between certain perfusion-based imaging features and activity of TFs STAT6 and TFAP2A. T2W texture features also correlated with STAT6 activity. STAT6 plays an important role in controlling cell migration and proliferation, while loss of AP2alpha protein expression, regulated by TFAP2A, is associated with PCa aggressiveness and progression. Therefore, radiomic features derived from MRI can predict csPCa and correlate with transcriptomic features.

Hectors et al. [74] demonstrated associations between mpMRI radiomic features and both histologic and genomic aggressiveness of PCa in a retrospective study of 64 patients with localized disease who underwent mpMRI before radical prostatectomy. They analyzed the index lesion (largest and highest Gleason score) from each surgical specimen. Significant correlations were found between mpMRI radiomic features (from T2WI, DWI, DKI sequences) and Gleason score, as well as with individual genes and transcriptomic signatures—in particular, Decipher (which predicts 5-year risk of metastasis after prostatectomy) (r = 0.40, *p* = 0.001) and PORTOS (predictor of postoperative radiotherapy response based on DNA repair genes) (r = −0.48, *p* = 0.002). Predictive models based on radiomics showed good performance in predicting Gleason score > 8 (AUC 0.72), and especially for Decipher score ≥ 0.6 (AUC 0.84), highlighting their utility in predicting tumor aggressiveness and metastasis risk.

Radiogenomics has also been used to explore the relationship between genetic alterations and hypoxia identified via MRI and their influence on cancer cell proliferation and progression [75]. Tumor hypoxia detected by MRI was associated with an aggressive MYC-driven gene expression program, suggesting a biologically active tumor with high progression risk. In contrast, mild hypoxic areas were associated with non-proliferative genes (CHEK1, EIF4G2, MARCKS, HDAC2, PTGES3).

PTEN is a tumor suppressor gene (located on chromosome 10q23), whose deletion or loss appears in various cancers and is associated with increased mortality and tumor aggressiveness in PCa [76,77,78]. Identifying PTEN loss may impact treatment selection. McCann et al. [76] analyzed 45 peripheral zone lesions from 30 patients who underwent mpMRI prior to radical prostatectomy, studying the relationship between PTEN expression and MRI parameters (ADC, T2WI, DCE). They found a weak but significant negative association between PTEN expression and Gleason score. Tumors with low PTEN expression had higher values of the DCE-derived parameter Kep, potentially reflecting increased vascular permeability and activity.

In a similar analysis by Switlyk et al. [78] in 45 patients, low PTEN expression correlated with greater tumor aggressiveness: higher Gleason score (*p* = 0.028), lymph node metastases (21% vs. 3%, *p* = 0.008), and extraprostatic extension (47% vs. 17%, *p* = 0.048). These tumors also showed lower ADC values, indicating higher cellularity and aggressiveness. However, no correlation was found between PTEN and DCE-MRI parameters like Kep.

A more recent study [77] examined the association of PTEN and ERG with both visible and non-visible PCa lesions on mpMRI. A retrospective analysis of 346 men who underwent mpMRI and immunohistochemical analysis of PTEN and ERG found that non-visible lesions had less PTEN loss and ERG positivity. These patients also had lower rates of high Gleason score, extraprostatic extension, seminal vesicle invasion, and lymph node metastases. However, PTEN and ERG did not significantly improve prediction of non-organ-confined disease or biochemical recurrence, suggesting limited added prognostic value beyond clinical and imaging variables.

Li P et al. [79] also investigated tumor visibility on mpMRI and its underlying biology, identifying four genes (PHYHD1, CENPF, ALDH2, GDF15) that predicted MRI visibility (AUC 0.86) and progression-free survival (PFS). These genes defined two groups with significantly different PFS outcomes (HR = 2.53 [1.55–4.11], *p* < 0.001; HR = 1.3 [1.04–1.63], *p* = 0.021), concluding that MRI visibility is associated with genetic features linked to poorer prognosis.

Beyond improving the prediction of clinically significant cancer, radiogenomics may help identify lesions with greater genomic burden and aggressive potential, facilitating selection of the index lesion for biopsy or focal therapy [74,80]. In this context, a prospective study [80] analyzed 46 targeted biopsies of both suspicious and non-suspicious areas on mpMRI and Ga-PSMA-PET/CT in 5 high-risk PCa patients. All patients subsequently underwent radical prostatectomy. The study introduced the concept of the “genomic index lesion,” defined as the biopsy with the highest number of large-scale CNAs (≥10 Mbp), considered a marker of tumor aggressiveness. Significant CNAs were found in 22 of 46 biopsies, with common losses at 8p, 13q, and 5q. The key finding was the strong correspondence between the radiologic index lesion and the genomic index lesion, which also matched the biopsy with the highest Gleason score. This suggests that advanced imaging techniques can guide localization of the most molecularly aggressive lesion [80] and help distinguish indolent from aggressive PCa, supporting decisions such as focal therapy, AS vs. radical treatment, or treatment intensification in multifocal tumors.

### 3.4. Liquid Biopsy and Imaging Correlation in Prostate Cancer

Liquid biopsy is an emerging, non-invasive tool that enables molecular characterization of tumors through circulating components in peripheral blood. In advanced prostate cancer, three main components stand out: circulating tumor DNA (ctDNA), circulating tumor cells (CTCs), and the androgen receptor splice variant AR-V7 (Table 3). These biomarkers provide valuable information on tumor burden, clonal heterogeneity, and therapeutic resistance, complementing conventional imaging techniques (CT, bone scan) and functional imaging (PSMA-PET).

Several studies demonstrate a significant correlation between radiologic tumor burden and the amount of biomarkers detected in blood [81,82,83]. Fonseca et al. [81] conducted a prospective observational study analyzing 491 patients with mCRPC. The study evaluated the correlation between plasma ctDNA fraction (ctDNA%), radiologic findings of tumor burden on conventional imaging (CT/bone scan), and clinical prognosis. They found that ctDNA% directly correlates with tumor burden as estimated by conventional imaging, with higher ctDNA% observed in patients with liver metastases (*p* < 0.001) and those with more than 10 bone lesions (*p* < 0.001). This may be due to ctDNA reflecting the number of active or necrotic tumor cells releasing DNA into the bloodstream, which is usually proportional to tumor volume observed by imaging (CT, bone scan, or PET) [81]. Additionally, ctDNA dynamics may reflect changes in metastatic patterns during clinical evolution in CRPC patients and anticipate clinical progression before PSA or imaging, correlating with response or resistance and providing added prognostic value [82,83].

Specifically, some prospective analyses have demonstrated a significant association between bone metastases and ctDNA dynamics (*p* = 0.003), highlighting the potential utility of ctDNA as a biomarker for monitoring patients with mCRPC and bone involvement [82].

On the other hand, Kluge et al. [84] prospectively evaluated the correlation between tumor burden measured by Ga-PSMA-11 PET/CT and plasma ctDNA levels in patients with advanced metastatic prostate cancer (both hormone-sensitive and castration-resistant). A positive but weak correlation was found between ctDNA and PSMA-PET in the overall patient group (*p* = 0.049), but not in hormone-sensitive disease (*p* = 0.837). However, the correlation was strong in the CRPC group (*p* = 0.007). Moreover, ctDNA levels were significantly higher in CRPC patients with greater tumor burden on PET, confirming that ctDNA increases with higher tumor burden, but only in CRPC patients. Conversely, high volume on PSMA-PET—rather than ctDNA (*p* = 0.12)—was significantly associated with poorer overall survival (*p* = 0.004), suggesting a stronger prognostic value [84].

Therefore, plasma ctDNA% complements imaging and molecular analysis in decision-making as an independent prognostic marker in mCRPC. It correlates with imaging and tumor burden biomarkers, though imaging and ctDNA provide distinct dimensions: while imaging reveals anatomical localization, ctDNA reflects the biological and genomic profile of the tumor. Their combination allows for better prognostic stratification.

However, current limitations of ctDNA should not be overlooked. For example, some tumors do not release sufficient tumor DNA into circulation, leading to false negatives in localized or low-volume disease. Furthermore, there is a lack of standardization in techniques and clinically validated thresholds [85]. Future research should aim to integrate ctDNA percentage with imaging, such as PSMA-PET, to improve quantification of total tumor burden.

Similarly, in the CRPC population, high CTCs counts are associated with extensive metastatic disease and worse prognosis. A prospective study of 181 mCRPC patients [83] found that detection of the AR-V7 variant in CTCs was linked to greater tumor burden on imaging, including visceral metastases (with high SUV on PET/CT) and a higher number of bone lesions. It was also associated with resistance to androgen receptor inhibitors like abiraterone or enzalutamide, indicating increased aggressiveness. The combination of AR-V7+ in CTCs and high imaging tumor burden was the strongest predictor of poor prognosis, meaning shorter radiographic progression-free survival and overall survival (*p* < 0.001).

Detection of AR-V7 in CTCs can also be helpful in selecting patients who are more likely to benefit from taxane-based chemotherapy. In the prospective study by Scher et al. [86] 161 men with mCRPC were analyzed. Among patients who were AR-V7–positive, those treated with taxanes had significantly longer PSA response durations, progression-free survival, and overall survival (median 8.9 vs. 4.6 months) than those who received androgen receptor inhibitors. These findings support the role of AR-V7 as a treatment-specific biomarker, suggesting that AR-V7–positive patients may derive greater benefit from taxane chemotherapy than from hormonal therapies such as abiraterone or enzalutamide.

Liquid biopsy may also be used to assess treatment response. In the review by Mayerhoefer et al. [87], liquid biopsy was found to be less sensitive than imaging in early stages, but more specific and capable of detecting therapeutic response earlier than conventional radiologic criteria (such as RECIST), offering a more sensitive and earlier monitoring tool in certain clinical contexts. In mCRPC, high ctDNA (>30) has been associated not only with greater risk of death and biochemical progression, but also with poorer response to treatment [81].

### 3.5. Integrative Models and Personalised Medicine

Integrative nomograms combining clinical, imaging, and biomarker data—particularly incorporating PSMA PET and mpMRI—represent a leading approach in personalized medicine for prostate cancer. These models enable refined risk stratification, optimized selection of candidates for invasive procedures, and individualized treatment strategies [54,88,89,90,91,92,93,94,95,96,97,98].

The Memorial Sloan Kettering Cancer Center (MSKCC) and Briganti nomograms (Table 4) are among the most validated tools for predicting lymph node invasion (LNI) and adverse pathology. Contemporary versions have integrated advanced imaging, notably mpMRI and PSMA PET/CT, as well as serum biomarkers, thereby enhancing predictive accuracy. Guideline-recommended thresholds for extended pelvic lymph node dissection (ePLND) vary—2% in the National Comprehensive Cancer Network (NCCN), 5% in the European Association of Urology (EAU), and 7% in the Briganti 2019 nomogram. Reported discriminative performance, expressed as the AUC, ranges between 0.70–0.80 for MSKCC, 0.70–0.80 for Briganti 2017, and 0.70–0.86 for Briganti 2019 [88,89].

A recent multicenter prospective study [88] demonstrated that incorporating PSMA PET findings significantly increased the AUC of Briganti 2017, MSKCC, and Briganti 2019 nomograms by 0.053, 0.065, and 0.052, respectively, while net benefit analyses indicated a 10–40% gain. Similarly, Gandaglia et al. [89] showed that applying a 5% PSMA PET-based cutoff would have spared 47% of ePLNDs compared with 13% using Briganti 2019, at the cost of missing only 2.1% of LNI cases.

Further integrative models have combined imaging and serum biomarkers. The Prostate Health Index (PHI), has emerged as a key biomarker in integrative models for prostate cancer detection and risk stratification. Multivariable models incorporating PHI alongside clinical variables (such as prostate volume, age, and family history) and imaging tools like mpMRI have demonstrated improved diagnostic accuracy and clinical decision-making, particularly in the context of PI-RADS 3 lesions, where diagnostic uncertainty is high. Some retrospective studies demonstrate that combining the PHI with PI-RADS scores improves diagnostic performance for PCa compared with either marker alone, enhancing sensitivity and specificity (81.3% and 68.1%, respectively). PHI correlates significantly with PI-RADS, Gleason score, and the number of positive biopsy cores, supporting its role in integrative models for risk stratification [99]. Other retrospective series of 931 patients with PI-RADS ≥ 4 lesions reported an AUC of 0.955 for a nomogram integrating PHI and PSA SUVmax, potentially allowing avoidance of 92% of biopsies while missing only 1.53% of csPCa cases. These results support the role of multiparametric, biopsy-free nomograms integrating PSMA PET, MRI-derived variables, and serum biomarkers [93].

Beyond serum and imaging biomarkers, tissue-based genomic classifiers have gained prominence for refining risk prediction. Decipher, Oncotype DX, and Prolaris are increasingly applied in both pre- and post-treatment settings to improve risk assessment and personalize management. These assays complement standard clinical and pathological parameters to guide treatment decisions, including eligibility for AS or adjuvant therapy after radical prostatectomy. In biopsy specimens, they refine stratification by evaluating tumor aggressiveness and predicting upgrading or progression, thus supporting the decision between AS and definitive treatment. Beyond its prognostic role for metastasis and biochemical recurrence, Decipher has demonstrated predictive utility in the AS setting, identifying men at higher risk of disease progression [100].

Alongside these developments, PSMA PET has emerged as a promising tool to complement genomic and serum biomarkers in AS. The PASPoRT trial [101] suggested that patients with intraprostatic SUVMax > 11 should not undergo AS without targeted biopsy, as higher uptake correlates with higher-grade cancer, while SUVMax < 5 strongly predicts stability. Preliminary data from the CONFIRM trial further support the role of PSMA PET in detecting MRI-occult, higher-grade lesions, potentially improving patient selection for AS [102].

The ongoing PRIMARY2 trial [54] is evaluating PSMA PET/CT in men with negative or equivocal mpMRI, using targeted biopsy only when PET is positive. The trial applies the PRIMARY score (Table 5), which integrates uptake pattern (focal vs. diffuse), zonal location, and SUVmax, with high specificity (100%) for csPCa when SUVmax ≥ 12. The score relies more on pattern than intensity, making it broadly applicable across different PET cameras and PSMA ligands.

Standardized frameworks such as PROMISE V2 [94] facilitate the integration of advanced imaging and biomarkers into nomograms. PROMISE V2 incorporates structured PSMA PET interpretation, the PRIMARY score, and molecular imaging TNM (miTNM) staging, along with response criteria such as PSMA-PET Progression (PPP) and Response Evaluation Criteria in PSMA-PET/CT (RECIP). These include variables such as total tumor volume (PSMA-VOL), lesion count, and PSMA expression score, all of which have prognostic relevance [95,96].

The PPP criteria (Table 6), initially developed for metastatic prostate cancer, focus on changes in individual lesions and are particularly suited for the limited disease burden typically observed in mHSPC. In contrast, RECIP (Table 6) provides a comprehensive framework for extensive disease by relying on PSMA-VOL rather than lesion count. This volumetric approach demonstrated excellent inter-reader agreement using semi-automated segmentation software (92%) and remained highly reproducible with purely visual volume estimation (83%) [95].

The standardized reporting framework of PROMISE V2 has potential utility across a range of indications including staging of high-risk patients, biochemical recurrence, and evaluation of suitability for ^177^Lu-PSMA radioligand therapy.

The authors additionally recommend quantifying tumor volume per organ system (miTNMa/b/c-nodal, bone, and visceral metastases), recognizing that metastatic site strongly influences biological aggressiveness. Increasing evidence supports PSMA-VOL as an independent predictor of overall survival [95].

Recent multicenter studies have shown that nomograms derived from PROMISE V2-based parameters outperform conventional risk scores. The PPP2 nomogram, developed by Karpinski et al. [97,98] from a cohort of 6128 patients, incorporates extrapelvic nodal, bone, and visceral metastases, PSMA expression score, and either lesion count (visual PPP2) or tumor volume (quantitative PPP2). Both versions achieved AUCs of 0.84 and a C-index of 0.80, stratifying patients into low-, intermediate-, and high-risk groups and surpassing NCCN and EAU risk models. This nomogram is now available for clinical use and should be prioritized as a tool in trial design.

A recognized limitation of PSMA-based systems is that decreasing PSMA expression may be misinterpreted as a therapeutic response. Moreover, in patients with limited disease, tumor volume alone may not be optimal for response assessment [95]. Interpretation is particularly complex in the context of recently initiated androgen deprivation therapy (ADT), as this can alter both disease extent and PSMA expression. In some cases, tumor shrinkage may be accompanied by increased PSMA uptake, potentially confounding treatment response assessment [95].

## 4. Discussion

Over the last decade, molecular imaging has transformed the diagnostic and therapeutic landscape of prostate cancer, redefining how disease is detected, characterized, and managed. PSMA-targeted PET has progressively supplanted conventional imaging as the foundation for diagnosis and staging, improving lesion detection and enabling more refined risk stratification across clinical settings. In the primary staging context, it is now routinely recommended for patients with high-risk localized or locally advanced disease, where it provides superior accuracy in defining tumor extent and metastatic spread. Its high specificity but moderate sensitivity for nodal metastases allows confident confirmation—but not exclusion—of lymph-node involvement [13,15]. Evidence from the proPSMA trial [2] and subsequent series highlights that its limited sensitivity reflects the sub-resolution scale of micrometastases, while biological heterogeneity in PSMA expression and variation in SUV thresholds contribute to diagnostic variability. As such, PSMA PET should not be interpreted in isolation but within validated, risk-adapted frameworks that integrate MRI and clinico-pathological parameters [5,18]. Its application in intermediate-risk unfavorable disease is expanding, though its cost-effectiveness and clinical impact in this subgroup remain to be fully defined.

In local T-staging, PSMA PET complements high-quality MRI, improving assessment of extracapsular extension and seminal vesicle invasion [17,21]. Within the prostate itself, it has shown excellent sensitivity for clinically significant disease, and the PRIMARY trial [16] confirmed that combining PSMA PET with mpMRI enhances diagnostic accuracy and negative predictive value, while reducing unnecessary biopsies. These findings, later corroborated by Mazzone et al. [5], demonstrate the complementary nature of both modalities, with PSMA PET–MRI offering greater precision for lesion detection and biopsy guidance. Nevertheless, technical and logistical barriers—scanner availability, acquisition time, and lack of standardized interpretation criteria—still limit its widespread clinical implementation.

Beyond primary staging, PSMA PET has become the reference imaging modality for biochemical recurrence (BCR), particularly in patients with previous definitive treatment who present with rising PSA. Its superiority over conventional imaging is most evident at low PSA levels. Meta-analyses [22,23] show a clear PSA-dependent gradient in detection, with higher positivity following radiotherapy compared with prostatectomy, reflecting differences in recurrence biology. However, reliance on the Phoenix definition (nadir + 2 ng/mL) may delay imaging and narrow the therapeutic window for effective salvage intervention [23]. This higher PSA threshold results in PSMA PET being performed at more advanced biochemical levels, naturally increasing the likelihood of detectable lesions. Moreover, recurrence after radiotherapy more often manifests as persistent intraprostatic disease or larger-volume locoregional relapse, both of which enhance detectability on PSMA PET. Therefore, later imaging driven by PSA criteria, greater residual intraprostatic tumour burden, and characteristic post-RT recurrence patterns could explain the higher positivity following radiotherapy. Prospective studies [24,25] have confirmed that PSMA PET can identify recurrence before biochemical or morphologic progression, enabling earlier and more targeted salvage strategies. Clinically, PSMA PET frequently alters management decisions—ranging from radiotherapy targeting to systemic therapy adjustment [26,27]—though a definitive impact on long-term survival outcomes remains to be established.

The integration of PET and MRI within hybrid scanners has further improved diagnostic precision, uniting molecular and anatomical data in a single examination. PET/MRI enhances local recurrence detection, particularly after prostatectomy or radiotherapy, and refines characterization of equivocal lesions where conventional imaging fails. By combining structural, functional, and molecular information, PET/MRI exemplifies how multiparametric imaging can contribute to more individualized care, although its high cost, scan duration, and limited accessibility currently restrict use to specialized centers.

The theragnostic paradigm has now been realized through PSMA-targeted radioligand therapy (RLT), directly linking molecular imaging to precision therapy. The β-emitter ^177^Lu-PSMA-617, validated in the pivotal VISION trial [34] and confirmed by TheraP [39], demonstrated significant improvements in radiographic progression-free and overall survival in patients with metastatic castration-resistant prostate cancer (mCRPC) who had previously received androgen receptor pathway inhibitors and taxanes. Similarly, the α-emitter radium-223 dichloride [30] provided the first survival benefit for patients with symptomatic bone-predominant mCRPC. These agents have established RLT as an effective and tolerable option in advanced disease, expanding therapeutic possibilities beyond conventional systemic therapy. Still, PSMA expression heterogeneity—regulated by androgen receptor (AR) signaling and influenced by tumor dedifferentiation—remains a critical determinant of efficacy. Prolonged AR inhibition may suppress FOLH1 transcription, reducing target availability, while transcriptional regulators such as HOXB13 may enhance PSMA expression [41]. Molecular alterations including RB1, TP53, MYCN, and AURKA are linked to neuroendocrine differentiation and resistance, underscoring the need for genomic stratification and dual-tracer imaging (PSMA + FDG) to identify discordant disease. Although the evidence is strongest in mCRPC, ongoing trials [45] are investigating the safety and efficacy of ^177^Lu-PSMA in hormone-sensitive and oligometastatic settings, where early results suggest delayed progression and deferred androgen deprivation.

MicroRNA-based approaches, particularly PSMA-targeted miR-34a, are emerging as experimental strategies in mCRPC, with preclinical studies showing selective tumour uptake and antitumour activity. Their current applicability remains limited by delivery and targeting challenges, but their ability to modulate resistance pathways and address molecular heterogeneity suggests a potential future role alongside radioligand therapy, especially in partially PSMA-negative or treatment-refractory disease work [46,47].

Artificial intelligence (AI) has emerged as a transformative adjunct in prostate cancer imaging, primarily within localized and intermediate-risk disease, where interpretation variability is greatest. The PI-CAI consortium [66] demonstrated that AI-assisted MRI reading can equal or surpass the diagnostic accuracy of general radiologists, particularly in detecting clinically significant prostate cancer and reducing variability in PI-RADS scoring. The upcoming PARADIGM trial [103] is expected to provide level 1 evidence for AI’s clinical utility, establishing its role as a decision-support tool. Beyond detection, explainable AI systems integrating MRI radiomics and clinical variables—such as PSA density or family history—are enhancing individualized risk prediction and biopsy triage. These systems improve diagnostic consistency, particularly in centers without subspecialized uroradiologists, representing a step toward standardized and equitable precision diagnostics.

Alongside these computational developments, radiogenomics represents a growing field at the intersection of molecular biology and imaging, offering a noninvasive bridge between phenotype and genotype [70]. In both localized and locally advanced disease, quantitative imaging features—such as apparent diffusion coefficient values, texture analysis, and perfusion metrics—have been correlated with key molecular alterations including PTEN loss, PI3K/AKT/mTOR signaling activation, hypoxia-driven MYC expression, and alterations in DNA-repair pathways (BRCA1/2, ATM) [71,72,73,78]. These correlations hold not only prognostic but also predictive potential, helping identify biologically aggressive lesions that may respond differently to radiation or systemic therapy. Spatial concordance between radiologic and genomic index lesions supports targeted biopsy and focal therapy strategies, improving diagnostic precision and minimizing overtreatment. From a clinical perspective, radiogenomics complements assays such as Decipher or Oncotype Dx by providing a whole-gland molecular map that contextualizes histologic findings and enhances treatment selection. While standardization of acquisition and feature extraction remains a limitation, AI-driven radiomic analysis and integration with multi-omic data are rapidly improving reproducibility, establishing radiogenomics as a pivotal tool for biologically guided decision-making.

In the same precision framework, liquid biopsy adds a dynamic, real-time molecular dimension to this framework, particularly relevant in advanced and metastatic disease. Analysis of circulating tumor DNA (ctDNA) and circulating tumor cells (CTCs) provides insight into tumor burden, clonal evolution, and therapeutic resistance. Detection of the AR-V7 splice variant identifies patients less likely to respond to androgen receptor inhibitors and more responsive to taxane chemotherapy [86], while ctDNA dynamics correlate with tumor volume and treatment response [81,84]. Although sensitivity is limited in localized disease and further technical standardization is needed, liquid biopsy complements imaging by capturing biological changes before radiographic progression, allowing earlier therapeutic adaptation.

Integrative predictive models uniting clinical, imaging, and molecular data have become essential tools for individualized management across disease stages. In localized and locally advanced prostate cancer, nomograms such as Briganti and MSKCC, enhanced with PSMA PET data, improve prediction of lymph-node invasion and guide surgical planning [89]. PHI-based models combined with mpMRI improve risk assessment in indeterminate lesions, while imaging-derived frameworks such as PROMISE V2 and PPP2 use PSMA-PET volumetric and molecular metrics to stratify prognosis and inform systemic therapy [97]. Despite their proven predictive accuracy, implementation in clinical practice remains limited by variability in access to advanced imaging and biomarkers, nonuniform data integration, and lack of automated tools.

Looking ahead, the future of prostate cancer management lies in the seamless integration of these advances—molecular imaging, theragnostics, AI, radiogenomics, and liquid biopsy—into adaptive, learning healthcare systems capable of capturing tumor evolution in real time. As these technologies mature, they will not only refine diagnosis and therapy but continuously adapt to each patient’s molecular trajectory. Although standardization, accessibility, and interoperability remain challenges, the convergence of these innovations is already reshaping clinical practice. Their collective utility now extends beyond research: these tools are actively enabling clinicians to personalize diagnosis, guide targeted therapy, and monitor response with unprecedented precision—marking a decisive step toward fully realizing precision medicine in prostate cancer.

## 5. Conclusions

PSMA-targeted imaging and theragnostics have redefined the diagnostic and therapeutic landscape of prostate cancer, enabling earlier detection, improved risk stratification, and biologically guided treatment across all disease stages. The integration of molecular imaging with artificial intelligence, radiogenomics, and liquid biopsy is optimizing patient selection, reducing unnecessary interventions, and guiding individualized therapeutic strategies that align management with tumor biology rather than anatomy alone. Taken together, advanced imaging and theragnostics are reshaping prostate cancer management, bridging molecular biology and clinical practice. Their continued evolution promises to refine patient stratification, improve clinical outcomes, and accelerate the transition toward precision oncology—marking the beginning of a new era of truly personalized care for patients with prostate cancer.

## Figures and Tables

**Table 1:** Global Methods Summary (own elaboration).

Clinical Question	Do novel molecular imaging modalities, theragnostics, radiogenomics, and liquid-biopsy biomarkers improve detection, staging, treatment selection, and monitoring compared with standard of care in patients with prostate cancer?
Population	(1) Humans; (2) Adults; (3) Patients with primary localized, recurrent, or advanced/metastatic prostate cancer
Intervention	PSMA PET/CT, PET/MRI, mpMRI, radiomics, radiogenomics; PSMA-targeted radioligand therapies (e.g., ^177^Lu-PSMA-617, ^225^Ac-PSMA); ctDNA, CTCs, AR-V7, multi-omics; AI-assisted imaging interpretation.
Comparison	Conventional imaging (CT, bone scintigraphy, MRI, [^18^F]FDG PET/CT); expert interpretation without AI support; standard clinical staging and monitoring protocols.
Outcomes	Primary: sensitivity/specificity, accuracy in T/N/M staging, detection rate, change-in-management, minimal residual disease detection (ctDNA/CTCs), radiographic/biochemical response, PFS, OS, therapy selection. Secondary: workflow impact, reproducibility, inter-reader agreement, cost-effectiveness, feasibility, safety
Study types	Prospective or retrospective clinical studies, clinical trials, systematic reviews, meta-analyses, pilot feasibility studies
Databases searched	PubMed, Embase, Cochrane Library
Search keywords	Prostate cancer AND (PSMA/PET-CT/PET-MRI/radiomics/radiogenomics) OR (radioligand therapy/theragnostic) OR (liquid biopsy/ctDNA/circulating tumor cells/biomarker) OR (artificial intelligence/integrative models) OR (personalized medicine).
Manual search	Screening reference lists and journal issues by hand
Inclusion criteria	Adult prostate cancer patients (localized, recurrent, or metastatic) Studies assessing advanced imaging (PSMA PET, mpMRI, PET/MRI), radioligand therapy, radiogenomics, liquid biopsy, or AI in diagnosis, staging, or treatment monitoring English/Spanish publications; years 2015–2025
Exclusion criteria	Preclinical/animal studies; case reports; conference abstracts without full text; editorials; narrative opinion pieces without patient-level or aggregated data.

Abbreviations: PSMA PET/CT = Prostate-Specific Membrane Antigen Positron Emission Tomography/Computed Tomography; PET/MRI = Positron Emission Tomography/Magnetic Resonance Imaging; mpMRI = multiparametric MRI; ^177^Lu-PSMA-617 = Lutetium-177–prostate-specific membrane antigen-617; ^225^Ac-PSMA = Actinium-225–prostate-specific membrane antigen; ctDNA = Circulating tumor DNA; CTCs = Circulating tumor cells; AR-V7 = Androgen receptor splice variant 7; T/N/M = Tumor/Node/Metastasis (staging system); AI = Artificial intelligence.

**Table 2:** Radiotracers in prostate cancer.

Tracer Type	Examples	Biological Target/Mechanism	Clinical Utility	Limitations
**PSMA ligands**	^68^Ga-PSMA-11 ^18^F-DCFPyL ^18^F-PSMA-1007	PSMA—transmembrane glycoprotein overexpressed on prostate cancer cells	Primary staging and restaging Detection of biochemical recurrence Selection/monitoring for PSMA-targeted radioligand therapy (^177^Lu-PSMA-617, ^225^Ac-PSMA-617)	Limited availability; heterogeneity of uptake; potential false positives (e.g., sympathetic ganglia, fractures, inflammatory lesions).
**Amino acid analogs**	^18^F-fluciclovine	Increased amino acid transport in malignant cells	FDA-approved for detection of recurrent disease after curative therapy	Less sensitive than PSMA PET, particularly at very low PSA levels.
**Choline-based tracers**	^11^C-choline ^18^F-choline	Increased phospholipid synthesis in cell membranes	Detection of recurrence and metastases	Inferior to PSMA PET; low sensitivity at PSA < 1 ng/mL; ^11^C short half-life limits distribution.
**Bone-specific tracers**	^18^F-NaF	Incorporation into hydroxyapatite during bone remodeling	Detection of bone metastases: high sensitivity and specificity for skeletal lesions; useful for therapy response in selected cases.	Restricted to bone imaging; no soft-tissue assessment. Less specificity than PSMA PET, uptake in benign osteoblastic processes.
**FDG**	^18^F-FDG	Glucose metabolism—elevated in aggressive or neuroendocrine variants	Complementary to PSMA PET in low-PSMA-expressing (dedifferentiated, neuroendocrine) or high-grade disease; prognostic value in mCRPC.	Poor sensitivity in typical adenocarcinoma; intense urinary excretion limits pelvic evaluation.

Abbreviations: ^68^Ga-PSMA-11 = Gallium-68–prostate-specific membrane antigen-11; ^18^F-DCFPyL = Fluorine-18–2-pentanedioic acid; ^18^F-PSMA-1007 = Fluorine-18–prostate-specific membrane antigen-1007; PSMA = Prostate-specific membrane antigen; PSMA PET = Prostate-specific membrane antigen positron emission tomography; ^18^F-fluciclovine = Fluorine-18–labelled fluciclovine; ^11^C-choline = Carbon-11– labelled choline; ^18^F-choline = Fluorine-18–labelled choline; ^18^F-NaF = Fluorine-18–sodium fluoride; ^18^F-FDG = Fluorine-18–fluorodeoxyglucose; ^225^Ac-PSMA-617 = Actinium-225–prostate-specific membrane antigen-617; PSA = Prostate-specific antigen; mCRPC = Metastatic castration-resistant prostate cancer.

**Table 3:** Biomarkers: ctDNA, CTC, and AR-V7.

Biomarker	Definition	Applications
ctDNA	DNA fragments released into the bloodstream by tumor cells	Reflects tumor burden (metastatic disease, high volume, proliferation). Identification of genetic alterations (AR, BRCA, TP53). Monitoring treatment response. Early detection of progression.
CTCs	Intact tumor cells detectable in peripheral blood	Indicate active disease. Allow for phenotypic and genomic studies.
AR-V7	Splice variant of the androgen receptor gene that produces a constitutively active receptor, independent of androgens.	Associated with resistance to androgen-axis inhibitors (abiraterone, enzalutamide). May guide treatment changes (e.g., toward chemotherapy).

Abbreviation: ctDNA = Circulating Tumor DNA; AR = Androgen Receptor; BRCA1/2 = Breast Cancer Gene; CTCs = Circulating Tumor Cells; AR-V7 = Androgen Receptor Splice Variant 7.

**Table 4:** Predictive performance of commonly used clinical nomograms for lymph node invasion (LNI) in prostate cancer, with and without PSMA PET integration.

Nomogram	Clinical Variables Included	AUC Without PSMA PET	AUC with PSMA PET
MSCKK	Total PSA, biopsy Gleason score, % of positive cores, clinical T stage, total number of cores.	0.71 (0.65–0.77)	0.77 (0.72–0.83)
Briganti 2017	PSA, clinical stage, biopsy Gleason grade group, % cores with highest-grade PCa, % cores with lower-grade PCa	0.70 (0.64–0.77)	0.76 (0.70–0.82)
Briganti 2019	PSA, clinical stage at mpMRI, Grade group at MRI-targeted biopsy, maximum diameter of the index lesion at mpMRI(mm), % cores with csPCa at systematic biopsy.	0.76 (0.71–0.82)	0.82 (0.76–0.87)

Abbreviations: AUC = area under the curve; MSCKK = Memorial Sloan Kettering Cancer Center; PSA = Prostate-Specific Antigen; PCa = Prostate Cancer; mpMRI = Multiparametric Magnetic Resonance Imaging.

**Table 5:** PRIMARY Scores. Adapted from Emmet et al. [16].

Score	Description
1	No dominant intraprostatic pattern and low-grade activity.
2	Diffuse TZ activity or symmetric CZ activity without focal uptake (including diffuse TZ activity with irregular focal uptake not clearly above background)
3	Focal TZ activity (visually greater than twice background TZ activity)
4	Focal PZ activity.
5	Any pattern with an SUVmax ≥ 12

Abbreviations: TZ = Transition zone; CZ = Central zone; PZ = Peripheral zone.

**Table 6:** Definitions of PPP and RECIP. Summary of criteria to assess disease progression or treatment response on PSMA PET/CT.

Criteria	Definition	
PPP	(1) Appearance of ≥2 new PSMA-positive distant lesions Or (2) Single new lesion accompanied by consistent clinical and/or laboratory changes (e.g., PSA, LDH, ALP, ECOG score) Or (3) ≥30% increase in size or uptake of an existing lesion with supporting clinical/laboratory evidence	
RECIP	Complete response	Absence of any PSMA-uptake on follow-up PET
	Partial response	≥30% reduction in PSMA-VOL without new lesions
	Progressive disease	≥20% increase in PSMA-VOL with new lesion
	Stable disease	Does not meet the above criteria

Abbreviations: PPP = PSMA-PET progression; LDH = Lactate dehydrogenase; ALP = Alkaline phosphatase; ECOG = Eastern Cooperative Oncology Group (performance status); RECIP (Response Evaluation Criteria in PSMA-PET/CT; PSMA-VOL = Prostate-specific membrane antigen–derived tumor volume.

## Data Availability

The original contributions presented in this study are included in the article; further inquiries can be directed to the corresponding author.

## Abbreviations

The following abbreviations are used in this manuscript:

ADC	Apparent Diffusion Coefficient
ADT	Androgen Deprivation Therapy
AI	Artificial Intelligence
AR	Androgen Receptor
ARPI	Androgen Receptor Pathway Inhibitor
AR-V7	Androgen Receptor Splice Variant 7
AS	Active Surveillance
ASCO	American Society of Clinical Oncology
AUC	Area Under the Curve
BCR	Biochemical Recurrence
BRCA1/2	Breast Cancer Genes 1 and 2
csPCa	Clinically Significant Prostate Cancer
CT	Computed Tomography
CTCs	Circulating Tumor Cells
ctDNA	Circulating Tumor DNA
DCE	Dynamic Contrast-Enhanced MRI
DNA	Deoxyribonucleic Acid
DRE	Digital Rectal Examination
DWI	Diffusion-Weighted Imaging
EAU	European Association of Urology
ePLND	Extended Pelvic Lymph Node Dissection
HR	Hazard Ratio
LNI	Lymph Node Invasion
mHSPC	Metastatic Hormone-Sensitive Prostate Cancer
mpMRI	Multiparametric Magnetic Resonance Imaging
MRI	Magnetic Resonance Imaging
MSKCC	Memorial Sloan Kettering Cancer Center
mCRPC	Metastatic Castration-Resistant Prostate Cancer
NCCN	National Comprehensive Cancer Network
PARP	Poly(ADP-ribose) Polymerase
PCa	Prostate Cancer
PET/CT	Positron Emission Tomography/Computed Tomography
PHI	Prostate Health Index
PI-RADS	Prostate Imaging Reporting and Data System
PFS	Progression-Free Survival
PLND	Pelvic Lymph Node Dissection
PPP	PSMA PET Progression
PSA	Prostate-Specific Antigen
PSMA	Prostate-Specific Membrane Antigen
PTEN	Phosphatase and Tensin Homolog
RECIP	Response Evaluation Criteria in PSMA-PET/CT
RLT	Radioligand therapies
RNA	Ribonucleic Acid
SPECT	Single-Photon Emission Computed Tomography
SRT	Salvage Radiotherapy
STAT6	Signal Transducer and Activator of Transcription 6
TFAP2A	Transcription Factor AP-2 Alpha
